# A single administration of the antibiotic, minocycline, reduces fear processing and improves implicit learning in healthy volunteers: analysis of the serum metabolome

**DOI:** 10.1038/s41398-020-0818-6

**Published:** 2020-05-13

**Authors:** Shi Yu Chan, Liliana Capitão, Fay Probert, Corinna Klinge, Sebastian Hoeckner, Catherine J. Harmer, Philip J. Cowen, Daniel C. Anthony, Philip W. J. Burnet

**Affiliations:** 1 Department of Psychiatry, University of Oxford, Oxford, UK; 2 Oxford Health NHS Foundation Trust, Oxford, UK; 3 Department of Pharmacology, University of Oxford, Oxford, UK; 4 Numares AG, Regensburg, Germany; 5 Present Address: Psychosis Neurobiology Lab, McLean Hospital, Belmont, MA 02478 USA

**Keywords:** Predictive markers, Human behaviour

## Abstract

Minocycline has shown therapeutic promise in pre-clinical animal models and early phase clinical trials for a variety of psychiatric disorders. Previous studies on minocycline have shown its ability to suppress microglia activity and reduce inflammatory cytokine levels, and its amelioration of depressive-like behaviour in animals and humans. However, the underlying mechanisms that lead to minocycline’s psychotropic effects are not clear. In this study, we investigated the psychological and biochemical effects of an acute dose of minocycline or placebo in 40 healthy adult volunteers. Psychological changes in emotional processing, implicit learning, and working memory were assessed. Plasma inflammatory markers, measured with enzyme-linked immunosorbent assays, and serum metabolites, measured with proton nuclear magnetic resonance combined with multi-variate analysis techniques, were also studied. Results showed that minocycline administration decreased fear misclassification and increased contextual learning, which suggested that reducing negative biases and improving cognition, respectively, may underlie the antidepressant actions of this agent. An examination of serum metabolites revealed higher levels of lipoproteins, particularly cholesterol, in the minocycline group. Minocycline also decreased circulating concentrations of the inflammatory marker C-Reactive Peptide, which is consistent with previous research. These effects highlight two important psychological mechanisms that may be relevant to the efficacy of minocycline reported in clinical trials, and also suggest a possible largely unexplored lipid-related biochemical pathway for the action of this drug.

## Introduction

Over one-third of people with depression do not respond to current, monoamine-based antidepressant treatments^1^. There is an urgent need, therefore, to develop novel therapies that target different mechanisms associated with the pathophysiology of mood disorders. There is now compelling evidence for the involvement of inflammation in depression. Serum levels of immune markers, particularly C-reactive protein (CRP) and inflammatory cytokines, have been shown to be raised in depressed subjects^2,3^, and both pre-clinical and clinical studies have demonstrated that pro-inflammatory cytokines induce cognitive and mood changes similar to those in depression^4,5^.

Minocycline, a broad spectrum tetracycline-derived antibiotic, has psychotropic actions in animals, and displays antidepressant properties when used as an adjunctive treatment for major depressive disorder^6^, bipolar depression^7^, psychotic depression^8^, and treatment-resistant depression^9^. Minocycline has also been suggested to have pro-cognitive effects^10^. Although the exact biochemical pathways underlying the central actions of minocycline remain elusive, its anti-inflammatory properties are proposed to play a key role. For instance, minocycline provided greater therapeutic benefit to bipolar patients with higher baseline levels of the pro-inflammatory cytokine interleukin-6 (IL-6)^7^, which may reflect minocycline-mediated inhibition of microglia activation and pro-inflammatory cytokines secretion^11,12^. In addition, minocycline has also been shown to have anti-oxidant, anti-glutamate excitotoxicity (possibly through modulating the kynurenine pathway), and neurogenesis effects^13^. Although these neuroprotective effects of minocycline are also poorly understood, the rapid advancements in analytical technologies may provide further insights on other mechanisms of action.

Untargeted metabolomic analyses with proton nuclear magnetic resonance (^1^H NMR) spectroscopy is a powerful, high throughput approach that has been used to explore the molecular signature of peripheral and central disease processes. For instance, measures of circulating amino acids and lipids in neurological conditions with overlapping clinical features has provided distinct metabolic profiles that could potentially improve the diagnosis of each disorder, and assist with the monitoring of their progression^14^. This technique, therefore, has the capacity to discern the biochemical components that underpin the actions of minocycline. Specifically, the profiling of blood metabolites following a single administration of minocycline will afford insight into the principal metabolic pathways that are immediately affected by the drug, and thence impact on brain function, but which are distinct from the antibiotic properties that follow its repeated intake.

The psychological mechanisms underlying the psychotropic actions of minocycline may be better understood through the assessment of its acute effects in the absence of clinical symptoms. Indeed, a single administration of antidepressants has been shown to reduce the processing of negative vs positive information^15–17^. In the neuropsychological model of antidepressant drug action, early changes in emotional processing towards a more positive valence are thought to precede and contribute towards improvements in mood^13^. Similarly, single doses of cognitive-enhancing drugs have also been shown to improve implicit learning^18,19^. To date, the psychological effects of minocycline that may underlie its clinical efficacy have not been examined.

The aim of the current study was therefore to test if a single administration of minocycline influenced: (1) emotional processing as a primary outcome, by using an emotional test battery that has successfully shed light on the psychological mechanisms underlying antidepressant action^17,20,21^; (2) cognitive function, as a secondary outcome, by using two implicit cognitive tasks and measures of attention and working memory that have detected acute effects of established pro-cognitive drugs^19^; and (3) immune responses by measuring changes in pro-inflammatory markers and metabolites.

Demonstrating concomitant minocycline-mediated changes in immune components and emotional and cognitive processing, when levels of pro-inflammatory cytokines are not raised, will provide further insight into the mechanisms underlying its psychotropic effects. Similarly, an examination of the changes in circulating metabolites following minocycline administration using ^1^H NMR metabolomics will inform on other potential pathways that are involved in its anti-inflammatory and/or central properties. Given the antidepressant and pro-cognitive effects of minocycline reported in previous clinical trials, we hypothesised that acute minocycline would increase the processing of positive vs negative information, and improve implicit learning. A parallel decrease in pro-inflammatory markers was also posited.

## Materials and methods

### Intervention

Minocycline (MEDA Pharma GmbH & Co. KG) was sourced from a pharmacy. Placebo were medicinal-grade lactose tables (HSC). A single dose comprised of two capsules, and was administered orally.

### Participants and study design

This double-blind, randomised, parallel and placebo-controlled study, received ethical approval by the Central University Research Ethics Committee (CUREC) (reference R50651) and has been registered with ClinicalTrials.gov (NCT03768557).

The study included 40 healthy volunteers (ages 18–55 inclusive) (Fig. S1), who provided written informed consent. A power calculation, to determine the sample size, was based on data showing that acute antidepressant administration reduced accuracy to detect fearful faces in healthy volunteers with an effect size of 1.09 (21). The minimum sample size required to detect changes in accuracy (difference between two independent means: two-tailed, alpha = 0.05, effect size = 1.09, power = 0.9) was calculated to be *n* = 19 per group.

Study design is summarised in Fig. S2. Participants had no current or past history of any psychiatric disorder as assessed using the Structured Clinical Interview for DSM-5^22^, no psychotropic medication in the past 3 months, no known hypersensitivity to tetracyclines, and not taken part in studies involving the same psychological tests. Further details on exclusion/inclusion criteria can be found in Supplementary Methods 1. Participants also completed questionnaires to assess verbal IQ (the National Adult Reading Test (NART)^23^), trait anxiety (the trait version of the STAI (STAI-T)^24^), personality (the Eysenck Personality Questionnaire (EPQ)^25^), and depressive symptoms (Beck Depression Inventory (BDI)^26^) at baseline.

On the testing day, participants were randomly assigned to either a single dose of minocycline (200 mg) or placebo (lactose). Randomisation was performed by a qualified researcher not involved in the study using the dedicated (free) software sealed envelope (https://www.sealedenvelope.com) (Supplementary Methods 2). Psychological testing commenced after a 2 h break (Fig. S2). Participants completed scales measuring state and mood (state version of the STAI (STAI-S)^24^, the positive and negative affective scale (PANAS)^27^, the Befindlichkeits scale (BFS)^28^, and the Bond–Lader visual analogue scale (VAS)^29^) at three time-points: (1) before drug/placebo administration, (2) before psychological testing, and (3) the end of study. At the end of the study, participants also completed an additional VAS measuring self-reported well-being and blinding. Finally, participants refrained from eating during the testing visit. Study time-points were chosen based on peak bioavailability of minocycline^30^ and feasibility. The whole testing visit lasted for around 5 h.

### Psychological tasks

Tasks are described in detail in Supplementary Methods 3.

#### Emotional test battery (ETB)

The ETB comprises five tasks measuring a participant’s bias towards positive or negative valence facial expressions and words, and consisted of the facial expression recognition task (FERT), emotional categorisation (ECAT), attentional dot probe, emotional recall (EREC), and emotional recognition memory (EMEM) task^16,17,31^. Outcome measures were accuracy, reaction time, and misclassifications. Percentage misclassification was calculated as the number of misclassifications in each emotion category divided by the total misclassifications per participant. For the FERT, a signal detection analysis was also performed to assess discriminability (*d*′), a measure of sensitivity, and response bias (*β*), a measure of conservativeness^32^. Finally, emotions in the FERT were defined as the six basic emotions (anger, disgust, fear, happy, sad, and surprise).

#### Cognitive tasks

Participants performed tests of implicit learning (contextual cueing task and priming task) and working memory (N-back task). The contextual cueing task measures the learning of spatial contextual information in the form of novel and repeated displays of symbols^33,34^. Differences between novel and repeated reaction times and accuracy were calculated for analysis. The priming task measured implicit memory, where prior exposure to a stimulus influences responses to the next stimulus^19^. Differences in the reaction times from the experimental and control conditions were calculated. Finally, working memory was measured by a letter variant of the N-back task as previously described^35^. Outcome measures were reaction time and accuracy.

### Blood collection

Blood was obtained from participants by venepuncture, at baseline (time-point 1) and around 4 h later at the end of study (time-point 3) (Fig. S2). Samples for serum (4 ml) and plasma (6 ml) were collected in appropriate vacutainer tubes and centrifuged to render samples acellular. Plasma and serum were then aliquoted and stored in a −80 °C freezer. Samples were excluded from analysis if insufficient volume was collected at either time-point (plasma: *N* = 3; serum: *N* = 2).

### Measurement of inflammatory markers and serum metabolomics

Enzyme-linked immunosorbent assays (ELISAs) were used to measure plasma cortisol, C-reactive protein (CRP), interleukin-6 (IL-6), interleukin-1β (IL-1β) (all from Abcam, Cambridge, UK) and tumour necrosis factor α (TNFα, RnD Systems, MN, USA) according to manufacturer’s instructions. Samples were diluted 1:5000 for CRP, and used neat for other measurements.

Serum metabolomics was conducted as previously described^14^. Serum from consenting participants (*N* = 18 × 2 time-points) was also sent to Numares AG (Regensburg, Germany) for further analysis of the lipoprotein populations and composition using the AXINON® lipoFIT® analysis platform. Metabolomics methods are described in detail in Supplementary Methods 4.

#### Pre-processing

All NMR spectra were imported into MestreNova (Santiago de Compostela, Spain) and each spectrum was manually phased, baseline corrected (Bernstein polynomial fit, order = 3), and referenced to an internal lactate (δ1.33). Spectra were then divided into 0.02 ppm “bins” and the integral of each bin calculated before further analysis. The water peak and noise regions were also removed for a final analysis of 175 bins.

### Statistical analysis

All researchers involved in the study remained blind to the treatment code throughout the entire study and data analysis duration. Unblinding only occurred after data analysis was completed.

#### Psychological tasks

Demographic data (except gender) and baseline characteristics were analysed with independent sample *t*-tests. All other measures and tasks were analysed with split-plot ANOVA where group (minocycline or placebo) was the between-subjects factor, and time, valence, and emotions were the within-subject factors. One participant in the minocycline group was excluded from the ETB analysis for abnormal responses (values > 3 × IQR).

#### Inflammatory markers

All data were expressed as a percentage change by normalising to individual baselines (where baseline = 100%) to address inter-participant variations, i.e., percentage change = (concentration of marker at baseline or time-point 3/concentration at baseline) × 100. The normalised values were analysed with split-plot ANOVA where group (minocycline or placebo) was the between-subjects factor, and time was the within-subject factor.

#### ^1^H-NMR metabolomics

All bins were expressed as a percentage change by normalising to individual baselines (where baseline = 100%) to address inter-participant variations, i.e., percentage change = (concentration of marker at baseline or time-point 3/concentration at baseline) × 100. The normalised values were used for further analysis. Three samples were determined to be outliers based on their Hotelling’s *T*^2^ value from a principal component analysis and excluded from further analysis.

##### Model building and validation

Orthogonal partial least squares discriminant analysis (OPLS-DA) models were built using in-house scripts (available upon request) in R 3.3.2^36^, the ROPLS package^37^ and a 10-fold external cross-validation scheme^14^ involving repeated testing of the models on independent data. A total of 100 iterations was conducted and mean values of accuracy, specificity, sensitivity, *Q*^2^, *R*^2^*X*, and *R*^2^*Y* of the ensemble of 1000 models were calculated. Model validation compared the genuine OPLS-DA models with those randomly permuted to test if the genuine models performed significantly better than random chance. For significant predictive models, the variable importance in projection (VIP) scores was calculated to identify the key bins that were important for the discrimination between classes.

##### Metabolite identification and direction of change

Metabolites were assigned to peaks in bins with high VIP scores through a combination of literature values^38^, reference to the human metabolome database (HMDB)^39^, and confirmed with two-dimensional (2D) correlation spectroscopy (COSY). The direction of change between groups was also determined by comparing means with independent samples *t*-tests. Where applicable, Bonferroni correction was used to correct for post-hoc multiple comparisons.

#### Multimodal analysis of significant variables

##### Discriminant analysis

A PLS-DA model was built from the significant variables from the different study components (see “Model building and validation”).

##### Correlation analysis

Correlations between variables were analysed with the Pearson correlation coefficient, and correlation matrix heat maps were produced in R 3.3.2.For split-plot ANOVA, equality of variance was tested with Mauchly’s test of sphericity, and when this assumption was not met, the Greenhouse–Geisser corrected value was reported. Non-parametric data were analysed with Mann–Whitney. All statistical tests were two-tailed and were run on SPSS (version 22), GraphPad Prism 5, and R 3.3.2. Data are expressed as mean ± SD.

##### General

Effect size was estimated using partial eta squared (ηp2).

## Results

### Baseline sample characteristics

There were no significant demographic differences between treatment groups, including gender, age, body mass index, and NART scores. The two groups were also well-matched at baseline for BDI, STAI-T, and EPQ (Table S1). There were no significant effects of treatment on mood scales at the different time-points (Table S2). Participants did not report any major adverse side effects of treatment, and no significant difference in wellbeing was reported between the minocycline and the placebo groups (Minocycline: 81.50 ± 14.83 vs Placebo −88.30 ± 12.44, *p* > 0.10). Finally, participants did not predict their group assignment when asked at the end of the study (*χ*^2^(2, *n* = 40) = 2.31, *p* > 0.10), which confirmed effective blinding.

### Emotional test battery (ETB) and cognitive tasks

#### FERT

##### Accuracy and misclassifications

**Fig. 1:**
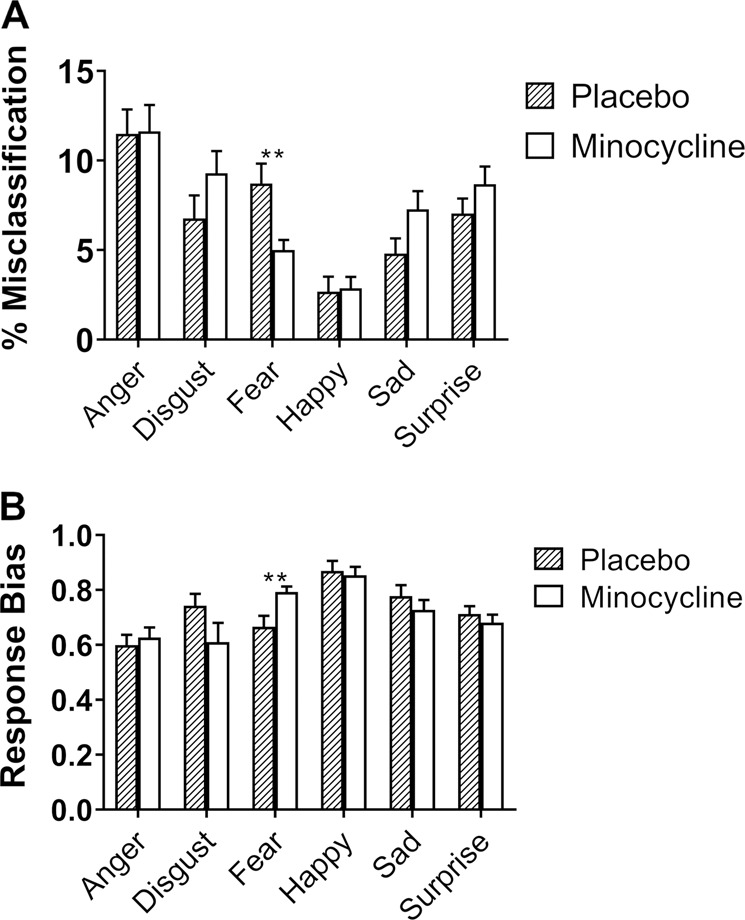
Effect of minocycline on emotional processing (FERT). **a** misclassification (expressed as % of total misclassification) and **b** response bias in FERT. Minocycline decreased misclassification of fearful expressions. A higher response bias score implies increased conservativeness when choosing fearful faces. Results are presented as mean+S.E.M. **p<0.01.

There was no significant interaction between emotion and group for total accuracy. However, there was a significant group × emotion interaction for percentage misclassifications (*F*_3.715, 137.446_ = 2.531, *p* = 0.047, *ηp*^2^ = 0.064). Pairwise comparisons revealed that participants in the minocycline group made significantly fewer fear misclassifications (mean difference −3.706, 95% CI −6.276 to −1.136, *p* = 0.006, *ηp*^2^ = 0.187) (Fig. 1a).

##### Signal detection theory: response bias and sensitivity index

A signal detection analysis was conducted by calculating response bias and sensitivity index. Response bias determines the likelihood ratio of participants choosing a particular emotion, and a higher value is associated with a more conservative response (decreased likelihood of choosing an emotion). There was a significant group × emotion interaction for the response bias (*F*_3.726, 137.874_ = 3.015, *p* = 0.023, *ηp*^2^ = 0.075). Pairwise comparisons revealed that participants in the minocycline group were more conservative when choosing fearful expressions compared to the placebo group (mean difference 0.127, 95% CI 0.035–0.219, *p* = 0.008, *ηp*^2^ = 0.174) (Fig. 1b). However, there was no significant difference in the sensitivity index between the two treatment groups for all six emotions.

#### Contextual cueing

##### Accuracy

**Fig. 2:**
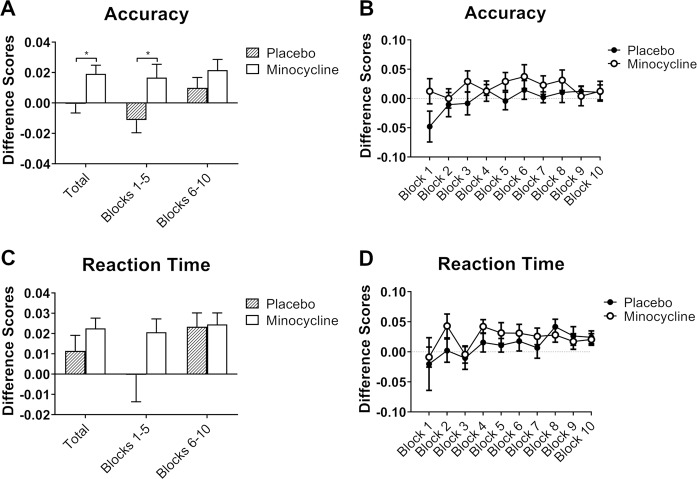
Effect of minocycline on implicit learning (Contextual cueing). **a**, **b** accuracy difference scores across all 10 blocks and across the first 5 blocks of the contextual cueing task. Reaction times (**c**, **d**) were similar between treatment groups. Results are presented as mean+S.E.M. *p<0.05.

There was a main group effect for accuracy averaged across all 10 blocks (*F*_1, 36_ = 5.917, *p* = 0.020, *ηp*^2^ = 0.141), with minocycline administration increasing accuracy (mean difference 0.020, 95% CI 0.003–0.036) (Fig. 2a, b). As an exploratory follow-up analysis, blocks were separated into two phases (blocks 1–5 and blocks 6–10). The difference in accuracy between the two groups was mainly driven by the first 5 blocks.

##### Reaction time

There was no significant main group effect for reaction time difference scores when these were averaged across all 10 blocks (Fig. 2c, d).

##### Other psychological tasks

There were no significant differences between treatment groups for ECAT, EREC, EMEM, the priming task, attentional dot-probe task, or the N-back task.

### Biochemical measurements

#### Inflammatory markers

**Fig. 3:**
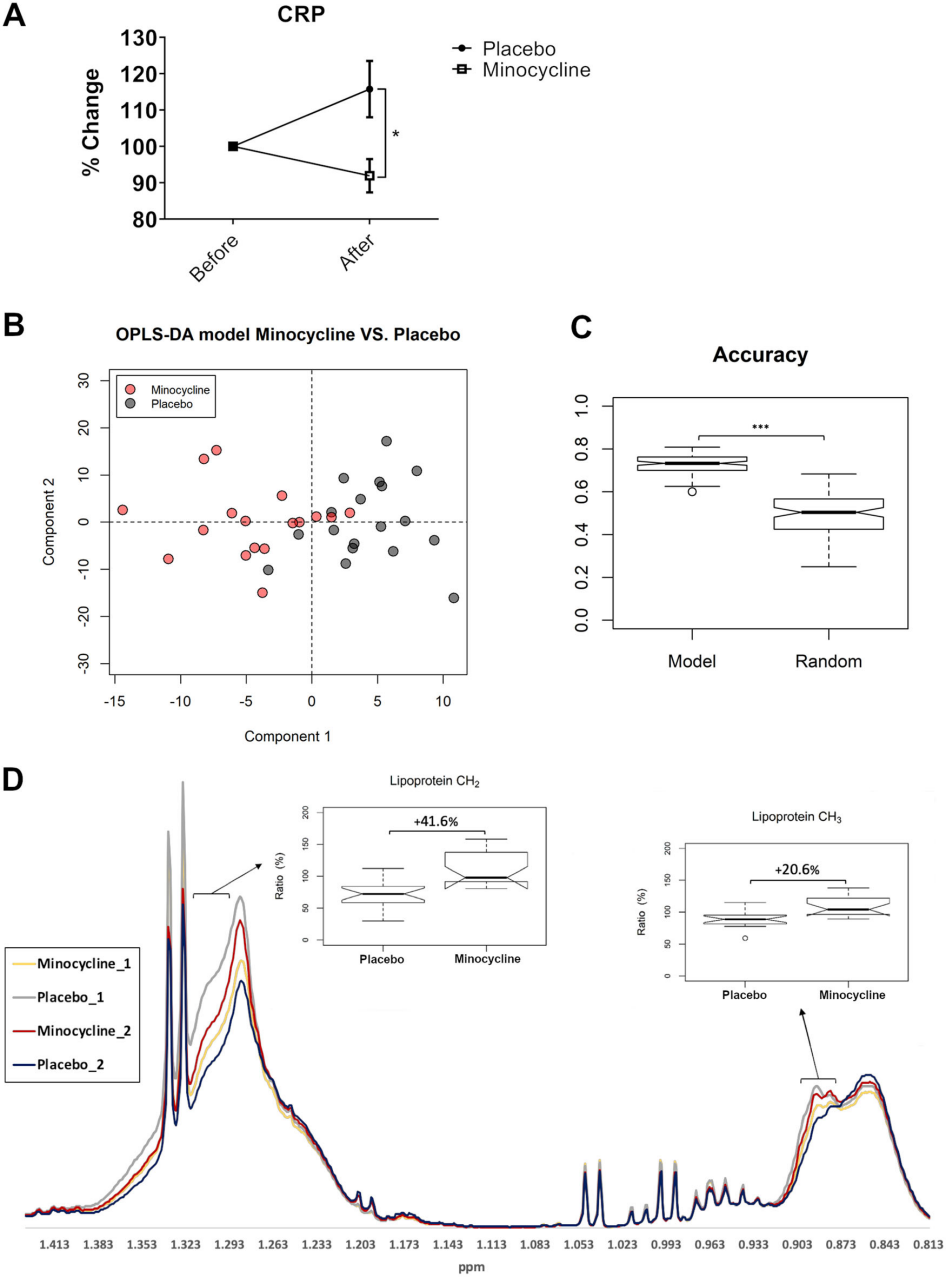
Effect of minocycline on plasma CRP and serum metabolites. **a** Plasma CRP levels before and after placebo/minocycline administration expressed as a percentage change of before. Results are presented as mean + S.E.M. **b** Scores plot of the OPLS-DA model separating samples by treatment group. **c** Accuracy of the Minocycline/Placebo models were significantly better than that of the ensemble of randomly permuted models. **d** Representative images showing that differences in lipoprotein peaks were the key variables for building the OPLS-DA models. Spectra expressed as average of treatment group. Boxplot hinges depict 1st quartile to median (lower) and median to 3rd quartile (upper). *p < 0.05; ***p < 0.001.

There was a significant time-point × group interaction for CRP (*F*_1,35_ = 6.781, *p* = 0.013, *ηp*^2^ = 0.162), where CRP levels were significantly lower in the minocycline group compared to the placebo group (mean difference −0.240, 95% CI −0.427 to −0.053, *p* = 0.013, *ηp*^2^ = 0.162) (Fig. 3a). Baseline CRP levels are reported in Fig. S1. No significant difference between treatment groups was found for cortisol (Fig. S4). Inflammatory cytokine levels in both treatment groups were low and largely beyond the detection level of the kits.

#### Metabolomics

Validation of baseline metabolite levels is reported in Fig. S5. Since participants refrained from eating between time-points, it was expected that metabolite levels would be lower at the end of the study. The percentage difference in the change in sum area (of all metabolite peaks) revealed that placebo, but not minocycline, decreased total metabolite levels (Placebo: −9.186%; Minocycline: +0.634%, *p* < 0.01) (Fig. S6).

To identify the metabolites driving the sum differences between treatment groups, untargeted OPLS-DA was used to build discriminant models (Fig. 3b). This ensemble of models was significantly more accurate (+23.2%, *p* < 0.001) at predicting sample-to-group assignment compared to the randomly permuted models (Fig. 3c). The sensitivity (+26.2%, *p* < 0.001), and specificity (+22.3%, *p* < 0.001) of the models were also significantly better than those randomly permuted (Fig. S7). Based on VIP scores, it was determined that minocycline administration prevented the decrease in levels of lipoproteins (Fig. 3d) that was observed in the placebo group.

#### Lipoprotein analysis

A total of 18 participants (Minocycline *N* = 11, Placebo *N* = 7) consented to having their samples sent for further lipoprotein analysis on the AXINON® lipoFIT® analysis platform. A power calculation of average lipoprotein peaks (Fig. 3d) showed that a sample size of 7/group had a power of 0.8 (difference between two independent means: two-tailed, alpha = 0.05, mean(1)74.97 mean(2)109.25, sd1(16) sd2(22)). Minocycline administration resulted in increased diameter of VLDL particles and the concentrations of: large VLDL (L-VLDL) particles, cholesterol in VLDL particles and small particles of LDL, and triglycerides in serum compared to placebo (Table 1). Post hoc correction (Bonferroni) revealed significant elevation in the cholesterol concentration in small particles of LDL (*p* < 0.01), and a trend increase in the mean diameter of VLDL particles in the minocycline group compared to placebo (*p* = 0.078).

**Table 1:** Summary of significant lipoprotein differences between minocycline and placebo expressed as the % ratio of time-point 3/time-point 1, measured by the AXINON® lipoFIT® analysis platform. LVLDL-p: concentration of large VLDL particles; VLDL-s: mean diameter of VLDL particles; VLDL-c: cholesterol concentration in VLDL class; LDL.C-c: cholesterol concentration in LDL subclass small particles; Sig: significance (*p* value); Adj Sig: significance adjusted for multiple testing with Bonferroni.

Metabolite	Minocycline (*N* = 11)		Placebo (*N* = 7)		Sig	Adj Sig
	Mean	SD	Mean	SD		
LVLDL-p	137.77	45.75	60.37	52.58	0.0216	0.5391
VLDL-s	104.01	3.75	95.39	6.39	0.0031	0.0781
VLDL-c	104.32	10.66	90.76	14.61	0.0422	1.0555
LDL.C-c	118.75	17.41	80.45	12.67	0.0002	0.0043
Triglycerides	103.62	25.71	63.47	21.84	0.0043	0.1072

### Multimodal analysis of significant variables

**Fig. 4:**
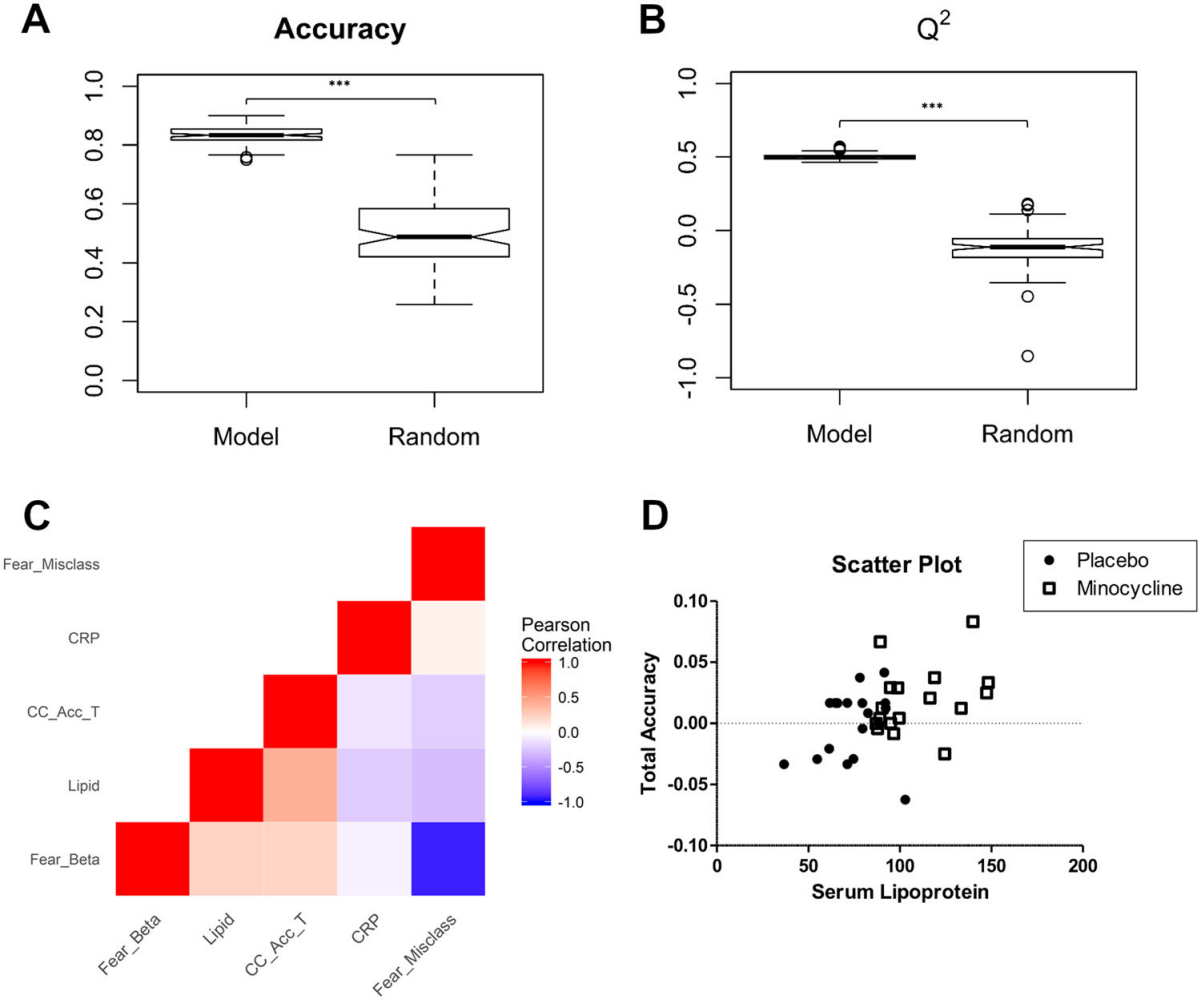
Multimodal analysis of significant variables. **a**, **b** Accuracy and *Q*^2^ values of the Minocycline/Placebo models were significantly better than that of the ensemble of randomly permutated models. Boxplot hinges depict 1st quartile to median (lower) and median to 3rd quartile (upper). **c** Correlations between different biochemical and behavioural outcomes displayed in a correlation matrix. **d** Scatterplot showing a positive correlation relationship between serum lipoprotein levels (averaged across significant bins) and total accuracy difference scores in the contextual cueing task. CRP: C Reactive Protein; CC_Acc_T: contextual cueing task total accuracy difference scores. ***p < 0.001.

Fear misclassification and response biasscores, contextual cueing accuracy difference scores, CRP levels, and serum lipoprotein levels were significantly affected by treatment. To validate these variables, models discriminating between treatment groups were built based on significant variables with 10-fold external cross-validation. The same predictors were used to build null models with the group labels permuted. Out of the 1000 models built (10-fold × 100 iterations), average predictive accuracy and *Q*^2^ was significantly better than the null models (accuracy: 83.2% vs 51.2%, *Q*^2^: 0.505 vs −0.092) (Fig. 4a, b).

Correlation analysis revealed that changes in serum lipoproteins had a positive correlation with contextual cueing accuracy difference scores (*r* = 0.407, *p* = 0.0154) (Fig. 4c, d). The serum lipoprotein variable was computed by averaging the values of all the significant serum lipoprotein bins.

## Discussion

To our knowledge, this is the first study to investigate the psychological and biochemical effects of a single dose of minocycline in healthy volunteers. Participants in the placebo group performed as expected in all tasks. Participants receiving minocycline showed a reduced number of fear misclassifications and were more conservative when choosing this emotion compared to the placebo group. The minocycline group also performed better in the implicit spatial contextual learning task. At a biochemical level, participants on minocycline showed decreased CRP levels as well as increased lipoprotein levels compared to the placebo group.

Decreased misclassification of fearful expressions by minocycline suggests that it reduced the processing of negative information. This is supported by a higher conservativeness score, where participants in the minocycline group were less likely to choose fear. A single dose of the antidepressant mirtazapine has also been shown to decrease the recognition of fearful faces in healthy volunteers^17^. Fearful expressions can be perceived as a threat and are inherently negative, and both antidepressant and anxiolytic drugs help remediate the negative bias and hypervigilance to threat that is seen in both anxious and depressive states^40^. Therefore, our current finding supports that minocycline, similar to antidepressants, modulates emotional processing that leads to downstream mood changes. Thus, reduced processing of negative information could be the mechanism that underlies the antidepressant-like effects of minocycline observed in early-phase clinical trials. Further research is nonetheless needed to clarify the exact processes by which minocycline may act to reduce negative bias, as the effect seen here was specific to fearful faces and did not extend to other negative facial expressions (like sad faces) or stimuli (negative words).

Minocycline also increased accuracy in the contextual cueing task, which indicates improved implicit learning. Visual contextual cues provide important information about the environment, giving meaning and influencing perception of what is observed^33^, and dysfunction in contextual learning has been observed in schizophrenia and depression^41,42^. It is also worth noting that, unlike other studies, we did not find effects of minocycline on executive functioning^10^ or attention/vigilance scores^43^. The reasons for these discrepancies are not clear, although the latter studies used repeated administrations of minocycline and also included schizophrenia patients with pre-existing cognitive impairments. Equally, since animal studies have shown that minocycline improves hippocampal function^44,45^, a brain region known to be involved in contextual learning, our findings may suggest that a single administration may influence the hippocampus more than cortical regions that mediate executive function. Additional research to corroborate our observations, therefore, should also include studies to test the primary neuroanatomical targets of minocycline.

We showed that CRP levels had decreased in the minocycline group compared to the placebo group after minocycline administration. It is possible that this is a result of a slight elevation in CRP levels in the placebo group (Fig. 3a). Given the relationship between acute stress and inflammation, it is also possible that the slight CRP elevation observed could be a result of acute stress from doing cognitively-taxing psychological tasks^46^. Nevertheless, actual CRP concentration showed that all participants had healthy CRP levels at both time-points (Fig. S3), and suggests that minocycline administration decreased or prevented the increase of CRP.

In addition to its anti-inflammatory properties, we have demonstrated that minocycline attenuated the fasting-mediated decrease in circulating metabolites observed in the placebo group (Fig. S6). Lipidomic analysis revealed that minocycline increased levels and/or cholesterol concentration in serum LVLDL, VLDL, LDL, and triglycerides. This suggests that the biochemical changes underlying minocycline’s psychotropic effects may also involve the acute phase response, as cholesterol levels are associated with acute phase proteins such as CRP. The combination of high CRP and low cholesterol have traditionally been linked to increased risk of coronary artery disease, but studies have also found high co-morbidity between cardiovascular disease and depression^47–49^. These findings are important as they highlight a previously unknown, complementary pathway through which minocycline may act.

Lipids are integral to membrane architecture and cell signalling, and changes in lipid components may affect synaptic function in the brain^50^. Indeed, there is evidence that major lipid species such as phospholipids, cholesterol, and sphingolipids are altered in patients with psychiatric disorders, and importantly, these lipids have been shown to be modulated by antidepressants in mice^51,52^. Furthermore, depression is associated with decreased serum LDL levels^53,54^. The current study also showed a significant positive correlation between lipoprotein levels and accuracy scores in the contextual cueing task, suggesting an association between lipids and implicit learning. This is supported by other studies that also show an association between serum lipids and cognitive function^55,56^.

While the exact nature of the associations between inflammation and lipids are unclear, it is worth noting that both inflammatory agents and chronic inflammatory disorders in humans reduce plasma cholesterol, LDL, and HDL levels^57^. Anti-inflammatory treatments that target pro-inflammatory cytokines, have also been shown to have mixed effects on lipids^58^. There is no doubt, therefore, that lipids and inflammatory markers interact, though further work is needed to understand the nature of these interactions. Nevertheless, given the importance of lipids in neural function, it is possible that both lipids and anti-inflammatory mechanisms have converging effects on synaptic transmission.

Finally, while this study focused on the anti-inflammatory properties of minocycline, it is also important to note that minocycline has been shown to have other effects, such as anti-oxidation effects and modulating the kynurenine pathway, that were not explored in this study. It is possible that the results found in this study could also be related to some of the other proposed minocycline pathways. Furthermore, as an antibiotic, minocycline has been shown to affect the gut microbiome^59^. However, gut microbiome changes that transmit to behavioural changes are unlikely to be detected in the current study as it involved only a single dose of minocycline with a short testing period (around 5 h). Nevertheless, given that minocycline affects multiple pathways and has been shown to have a variety of effects in different disease models^59,60^, we would like to note that our observations do not represent all the possible properties/actions of minocycline.

### Limitations

There are some limitations to the present study. First, while ^1^H NMR is a robust and rapid method for metabolomic analyses, its resolution of lipids is poor. Lipids appear on ^1^H NMR spectra as broad peaks, and it is difficult to identify exactly which lipid species are changing. Further analyses of minocycline–lipid interactions should therefore be performed using high-resolution mass spectrometry. Second, the focus of this study was to investigate the psychological and biochemical mechanisms of minocycline, in the absence of the confounding effects of clinical symptoms. Although this is desirable in mechanistic studies, the clinical relevance of our findings is unknown. Therefore, future studies should investigate the effects of minocycline in depressed subjects to determine if early changes in emotional processing lead to improved mood. Furthermore, inflammatory cytokine levels in this sample of healthy individuals were often below the detection limit of the assays and so the anti-inflammatory effect of minocycline could only be suggested by the more abundant CRP. Finally, our observations do not inform on the action of minocycline when administered repeatedly, as would be the case in a clinical setting. Separate studies are required to confirm whether acute and chronic minocycline intake share similar psychological, immune, and metabolic effects.

## Conclusion

In conclusion, a single dose of minocycline is sufficient to induce detectable psychological changes. Reductions in fear misclassification suggest that minocycline affects emotional processing in a way that implies an antidepressant-like action. The contextual cueing task data also suggest that minocycline improves implicit learning. These effects are important as they elucidate key psychological and cognitive mechanisms that may precede and contribute to the beneficial effects of minocycline on mood and cognition as reported in previous clinical trials. This study also supports an anti-inflammatory property of minocycline. Lastly, the effects on lipid metabolism highlight a new biochemical pathway that might underlie the action of minocycline, and which warrants future investigation.

## Disclaimer

The views expressed are those of the authors and not necessarily those of the NHS, the NIHR, or the Department of Health.

## Supplementary information

Supplementary information
