# Supplementary material for: A single administration of the antibiotic, minocycline, reduces fear processing and improves implicit learning in healthy volunteers: analysis of the serum metabolome
Source: Transl Psychiatry. 2020 May 13;10:148. doi: 10.1038/s41398-020-0818-6 (PMC7220900; doi:10.1038/s41398-020-0818-6)
Supplement: Supplementary file 1 — Supplementary information [file 41398_2020_818_MOESM1_ESM.pdf]

Supplementary Methods

Item 1: Exclusion/Inclusion Criteria

Participants who were outside the age limit (18-55), had a current or past history of any psychiatric disorder, had any medical contra-indication, were taking medication that would interfere with minocycline or cause any contra-indications, had known hypersensitivity to tetracyclines or to any of the excipients in the minocycline or placebo capsules, were currently pregnant or breastfeeding, were lactose intolerant, smoked more than 5 cigarettes a day, had a body mass index (BMI) outside the range of 18-30 inclusive, or were dyslexic were excluded. A washout period was also enforced for participants who had previously participated in studies involving the use of medication (3 months), brain stimulation (1 month), or who had recently used psychotropic drugs (3 months). To avoid learning effects, participants who had taken part in studies involving the same battery of emotional processing tasks were also excluded. Finally, to ensure that female participants would not be unduly influenced by hormonal changes, testing sessions during the pre-menstrual period was avoided.

The full list of medical conditions assessed for include:

**Medical problem/condition**
History of seizures/epilepsy
History of blackout, faint or dizziness when standing
Hepatic, renal or urinary (*Kidney*) dysfunction
Liver dysfunction (i.e. hepatitis or any chronic conditions)
Cardiac or respiratory problems (i.e. Palpitations/racing heart; Asthma; high or low blood pressure; lung pains; persistent cough)
Endocrine dysfunction/hormonal imbalance (progesterone, estradiol, testosterone, prolactin excess/deficiency), diabetes, thyroid problems
Autoimmune disorders (like Crohn's disease, arthritis, etc.)
Allergies (i.e. especially lactose intolerance)
History of bleeding disorders (i.e. cutaneous bleeding)
Skin and subcutaneous tissue disorders (i.e. rashes, urticaria, purpura)
Persistent headaches/migraines
History of visual disorders or difficulties
Dyslexia or any other reading impairments
GI problems (e.g., irritable bowel syndrome or any stomach problem)
Any past adverse reactions to any medication (especially antibiotics - tetracyclines)

If participants answered yes to any, a medical doctor was consulted to ensure participants could be included in the study.

Before the testing visit, participants were also asked not to consume alcohol the night before the testing visit, not to engage in intense exercise the morning of the visit, not to cycle the day of the visit, and not to smoke during the entire testing visit.

Item 2: Randomization and Blinding

Randomisation was performed by a qualified researcher not involved in the study using the dedicated (free) software sealed envelope (https://www.sealedenvelope.com). The randomisation programme included a minimisation algorithm to ensure balanced allocation of participants across the treatment groups by sex, and used a block design of 4. Allocation ratio was 1:1 for treatment (minocycline vs. placebo) and 1:1 for sex (male and female). In total, 40 participants were randomised; 20 were allocated to minocycline (10 male and 10 female) and 20 to placebo (10 male and 10 female).

The code was stored in a locked filling cabinet in the Neurosciences Building at the Department of Psychiatry, Warneford Hospital, Oxford, England. Both minocycline and placebo were encapsulated using identical capsules, and the individual single doses were stored in sealed envelopes labelled with the corresponding study code. All researchers involved in the study remained blind to this code throughout the entire study duration (May 2017 to February 2018) and data analysis (after February 2018). The study stopped when the sample size was reached (N=40). Unblinding only occurred after data analysis was completed.

All the study sessions were conducted at the Department of Psychiatry, Warneford Hospital, Oxford, England.

Item 3: Description of Psychological Tasks

Facial Expression Recognition Task (FERT): The FERT measured a participant's ability to differentiate between 6 basic emotions (anger, disgust, fear, sadness, surprise, and happiness) and neutral, presented as images of facial expressions. The images were taken from the Pictures of Affect Series [1] and shown at varying intensities in 10% gradations ranging from 0% (neutral) to 100% (full emotion). A total of 250 (10 individuals contributing a total of 4 examples of each emotion at each intensity, plus a neutral expression for each individual) randomized images were presented on a computer monitor for 500 ms per image. Analysis of the data involved looking at accuracy scores, reaction time, and misclassifications. Accuracy scores comprised of total accuracy (total number of correct responses for each emotion). Misclassifications are defined as the number of responses when the participant chose a facial expression when it was actually an expression of a different category. A signal detection analysis was also performed to assess discriminability (d'), a measure of sensitivity, and response bias (β), a measure of conservativeness. A higher β score is associated with a more conservative score, implying a decreased likelihood in choosing an emotion. Grier calculated these measures using the equations $d' = 0.5 + ((y-x)(1+y-x)/4y(1-x))$ and $\beta = (y(1-y)-x(1-x))/(y(1-y)+x(1-x))$ where x = probability of false alarms (number of false alarms/total number of distractors) and y = accuracy (number of hits/number of targets). [2]. For all analyses, emotions were defined as the 6 emotions (anger, disgust, fear, happy, sad, surprise) and neutral was considered as baseline and not included in the analyses.

Emotional Categorization (ECAT), Emotional Recall (EREC), and Emotional Memory (EMEM): For the ECAT, participants were presented with 60 personality characteristic words on a computer screen for 500 ms. The words [3] were selected to be positive or negative in nature (30 each), and were matched for length, frequency and meaningfulness. Participants were asked to think of the words as though overhearing someone describing them with that characteristic, and their task was to determine if they would like or dislike to be described in that manner. Analysis of the data involved looking at the number of classifications, and response times for correct identification. The EREC is a surprise recall task where participants were given 2 minutes to write down as many characteristic words as they could remember from the ECAT. Emotional biases in memory was measured as the relative recall of positive versus negative words. Finally, in the EMEM, participants were presented with a series of personality characteristic words on a computer screen. Half of the words presented were positive and negative words previously seen by participants in the ECAT, while the other half were novel distracter words. Participants were asked to determine if they had previously seen the word (familiar) in the ECAT or not (novel). These tasks measured emotional biases in memory, and data analysis involved looking at the accuracy and response time of the recall, and the valence of the recall (i.e. whether positive information was remembered better).

Attentional Dot Probe: Participants were shown a display where a pair of faces would appear (one on the top half of the screen and the other at the bottom half), followed by 2 dots that were either in vertical or horizontal orientation in the location of one of the faces. Participants were asked to respond by pressing a key corresponding to the orientation of the dots. There were three possible emotional pairings of the faces shown – 1. Neutral-neutral 2. Happy-neutral 3. Fearful-neutral. If participants had an attentional bias towards negative stimuli, they would respond faster when the dots appear in the location previously occupied by a fearful face than when the dots appeared in the other location. The emotional faces (happy and fearful) appeared in both locations at equal frequency. The faces shown were photographs of 20 individuals with different facial expressions taken from the JACFEE/JACNeuF sets of facial expressions [4]. Data was analysed by calculating attentional vigilance scores for each participant from mean reaction times. Namely, comparing between congruent trials, where probes appears in the same location as the emotional face, and incongruent trials, where probes appeared in the opposite position. Positive scores would imply an attentional vigilance towards the emotional stimuli.

Cognitive Cueing: This task, adapted from Chun & Jiang (1998), measured the learning of spatial contextual information [5, 6]. Participants were presented with a display of an array of 11 distracter "L"s with a single target "T" on a computer screen. Participants were then asked to locate the T and press a key indicating if it was tilted to the left or to the right. In each trial, a central fixation dot is shown for 500 ms, followed by the display array for 700 ms or until participant response. There are 48 trials in a block, with the display array a repeat of a previously presented array in half the trials, and novel display arrays for the remaining half. Altogether, participants are asked to complete a practice block (where the "T" is in a different colour from the distracters) and 10 actual blocks where participants received feedback at the end of each block (percentage accuracy and time taken). Data was analysed by calculating difference scores from the reaction time and accuracy for repeated arrays versus reaction time and accuracy for novel arrays. Reaction times greater than 2000 ms were not recorded.

Priming: This task measured implicit memory, where prior exposure to a stimulus influences responses to the next stimulus [7]. Participants were asked to locate an "X" that would appear at one out of 4 possible locations on the screen, and press a corresponding location key on the keyboard. A distracter "O" would also be shown at a second location. Each trial starts with the presentation of a fixation cross for around 1500 ms, followed by a priming display. Participants will then be presented with a second shorter fixation cross for around 400 ms, and finally a probe display. Participants will have to respond to both the priming and probe display, and both displays remain on screen until a response is recorded. The locations of the "X" and "O" within the two displays in a trial result in 4 conditions – 2 experimental conditions (repetition priming and negative priming), and 2 control conditions (imbalance and control). There are a total of 192 trials (48 trials per condition), presented to participants in 3 blocks with a 15 second break in between blocks. Data was analysed by calculating difference scores from reaction times from the 2 experimental

conditions compared to the control condition. Only trials with correct responses to the prime display were included in the analysis.

N-back: This letter variant of the N-back task measures working memory [8]. Participants were shown a series of letters, and asked to respond by clicking either the key "same" or "different" depending on the task condition. This task had four conditions. In the 0-back condition, participants were asked to select "same" when the letter "X" appeared, and different for all other letters. In the 1-back condition, participants were asked to compare each letter with the one before and select "same" if the current letter was the same as the one before. In the 2-back condition, participants were asked to compare between the current letter and the letter shown two places ago. Finally, in the 3-back condition, participants were asked to compare between the current letter and the letter shown three places ago. Each trial starts with a screen stating the condition, followed by a central fixation cross. The end of the trial is indicated by the appearance of a central fixation cross. Data analysis for this task involved looking at reaction time and accuracy.

Item 4: ^{1}H NMR metabolomics

Serum metabolomics was conducted as previously described [9]. Serum samples were defrosted on ice and centrifuged at 17,000 xg for 5 min at 4 °C. An equal volume (150 µL) of serum were aliquoted into a fresh tube and diluted to 600 µL with 74 mM sodium phosphate buffer D_2O (pH 7.4) All NMR spectra were acquired using a 700-MHz Bruker AVII spectrometer operating at 16.4 T equipped with a ^{1}H (^{13}C/^{15}N) TCI cryoprobe. Sample temperature was stable at 310 K. ^{1}H NMR spectra were acquired using a 1D NOESY presaturation scheme for attenuation of the water resonance with a 2 s presaturation. A spin-echo Carr-Purcell-Meiboom-Gill (CPMG) sequence with a τ interval of 400 µs, 80 loops, 32 data collections, an acquisition time of 1.5 s, a relaxation delay of 2 s, and a fixed receiver gain was used to suppress broad signals arising from large molecular weight plasma components. CPMG spectra provide a measurement of small molecular weight metabolites and mobile side chains of lipoproteins in the plasma sample and were used for all further analysis. 750 µL of serum from consenting participants (N = 19 x 2 time-points) was also sent to numares AG (Regensburg, Germany) for further analysis of the lipoprotein populations and composition using the AXINON® lipoFIT® analysis platform.

Pre-processing: NMR spectra were imported into MestreNova (Santiago de Compostela, Spain) and each spectrum was then processed manually with phase 0 (PH0) correction, baseline correction (Bernstein polynomial fit, order = 3), and referencing to an internal standard (Lactate referenced to δ1.33). The individual spectrums were stacked, and binned (sum method, width of each integral region = 0.02 ppm). Binning refers to a function where the whole spectrum is divided into bins of equal width, and all the peaks in each bin is integrated to obtained a value representing the area of all the peaks in a bin. Binned values were then exported as a spreadsheet (.csv) for further analysis. The water peak and noise areas were also removed for a final number of 175 bins included for further analysis.

As there were two time-points for each participant, the percentage change between time-points for each bin was calculated by taking the ratio of time-point 2 divided by time-point 1 and multiplying the ratio by 100.

Preliminary exploratory analysis: The original bin values and ratios were then imported into SIMCA (Umetrics, Sweden). Principle component analysis (PCA) scores plot were used to identify outliers as well as inter-individual variation. Pareto scaling was used for original values, whereas UV scaling was used for the percentage ratios. Supervised multi-variate analysis was conducted with orthogonal partial least squares discriminant analysis (OPLS-DA), where samples were separated by treatment.

Model building and validation: OPLS-DA models were built in R 3.3.2 [10] using the ROPLS package [11] and a 10-fold cross validation scheme [9] involving repeated testing of the models on independent data. A total

of 100 iterations was conducted and mean values of accuracy, specificity, sensitivity, Q^2, R^2X, and R^2Y of the models were calculated and presented as boxplots.

Validation of the models was conducted where the genuine OPLS-DA models were compared with models generated by randomly permuting the class assignments to see if the genuine models performed significantly better than random chance (determined by the accuracy, specificity, and sensitivity). For significant, predictive models, the variables in projection (VIP) scores were calculated to identify the key bins that were important for the discrimination between classes. Key bins were identified by sorting all bins in descending order of VIP score and plotting a graph of bins (x axis) against VIP scores (y axis). Bins which result in the largest decrease in accuracy when removed from the model (steepest gradient in the VIP plot) are the most important discriminatory variables, hence the inflection point in the graph (the point at which the graph first plateaus) is taken as the threshold VIP value; all bins with VIP scores above this value are considered significant for driving the discriminatory model.

<u>Metabolite identification and direction of change:</u> Metabolites were assigned to peaks in bins with high VIP scores through a combination of literature values [12], reference to the human metabolome database (HMDB) [13], and confirmed with two-dimensional (2D) correlation spectroscopy (COSY). The direction of change between groups was also determined by comparing means in SPSS (independent samples T-test). When assumptions for parametric testing were not met, the non-parametric test Mann-Whitney was used instead. Where applicable, Bonferroni correction was used to correct for multiple comparisons.

Supplementary Figures and Tables

Fig S1: CONSORT Recruitment flow diagram

Recruitment flow diagram (based on CONSORT)

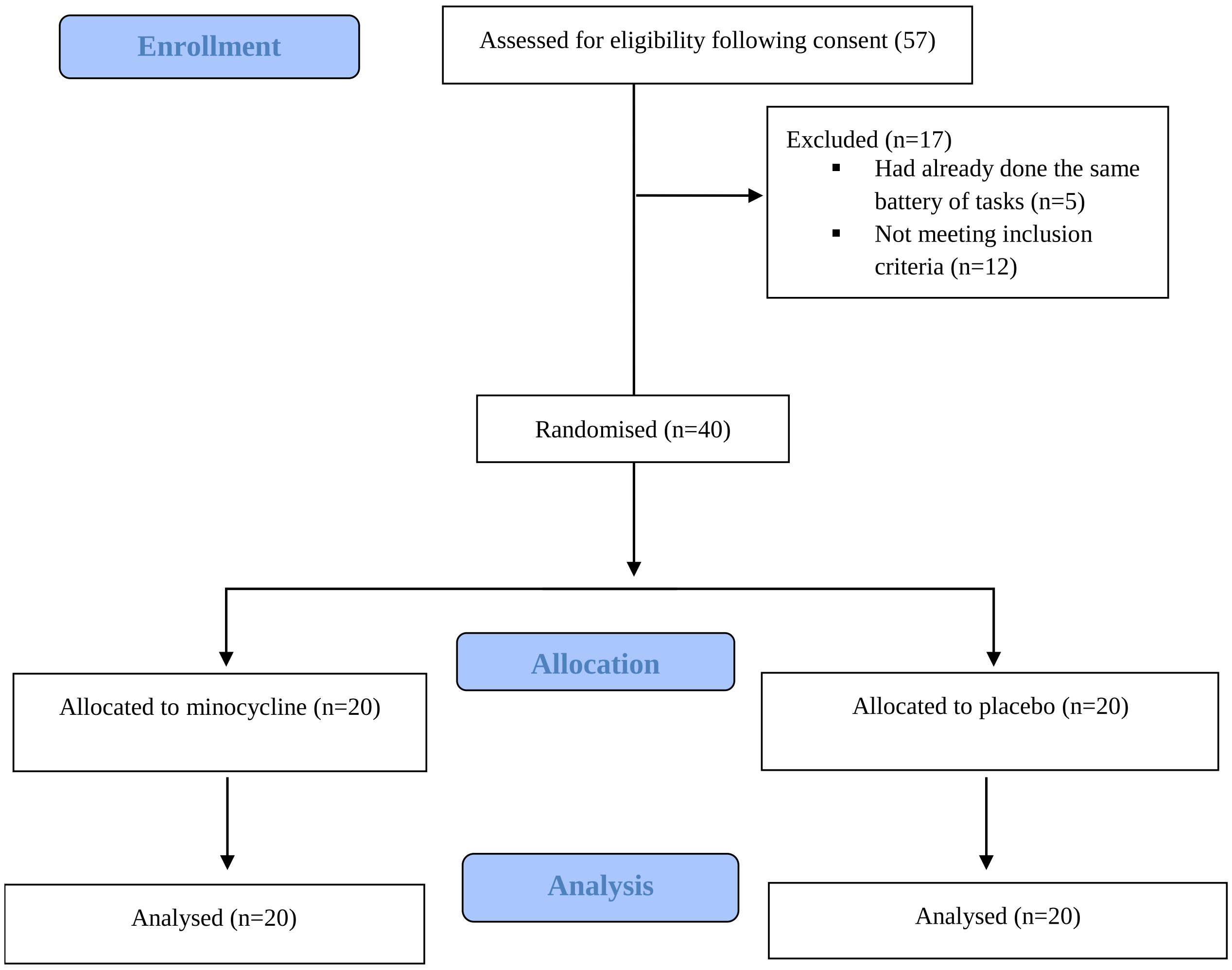

Fig S2: Study Design

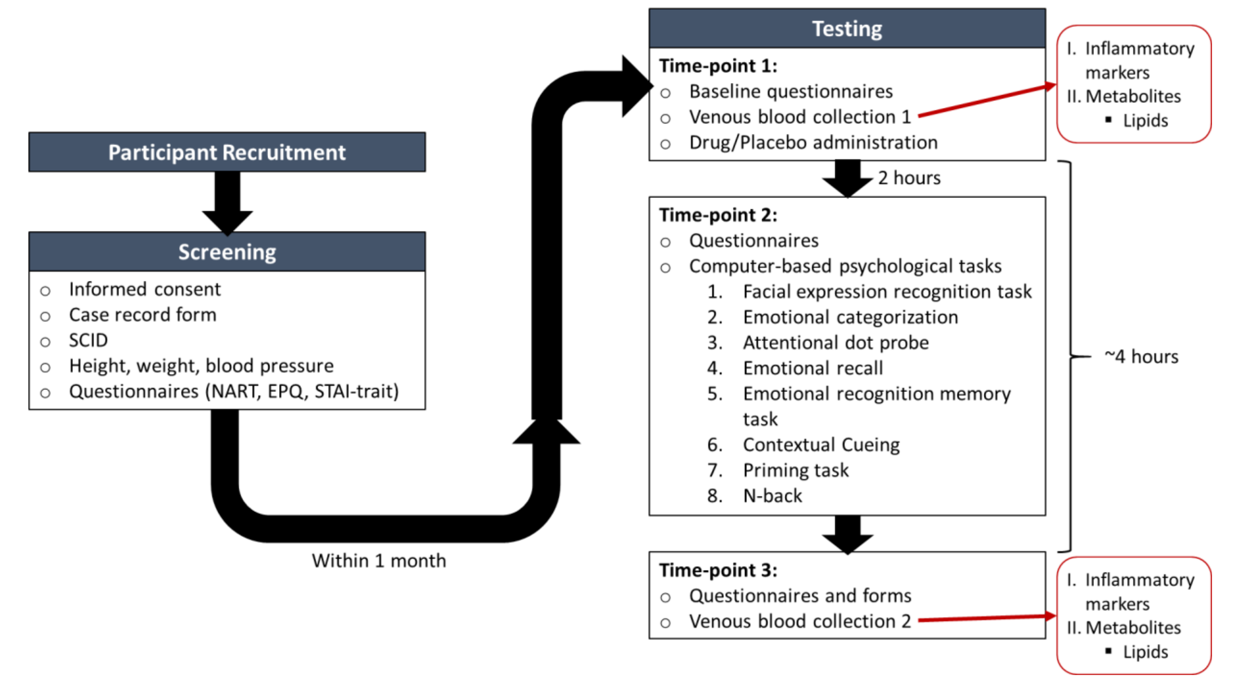

Fig S2 Study Design: Participants undergo a screening visit and a testing visit. The testing visit is composed of three time-points – 1. Before minocycline/placebo administration; 2. Psychological testing; 3: End of study. Venous blood samples are first collected before minocycline/placebo administration, and a second time approximately four hours later at the end of the study visit. The testing visit lasts for around 4.5 hours. Blood samples are used for the measurement of inflammatory markers and metabolites, and a subset of samples from consenting participants were sent for further lipid analysis.

Fig S3: Baseline CRP Validation

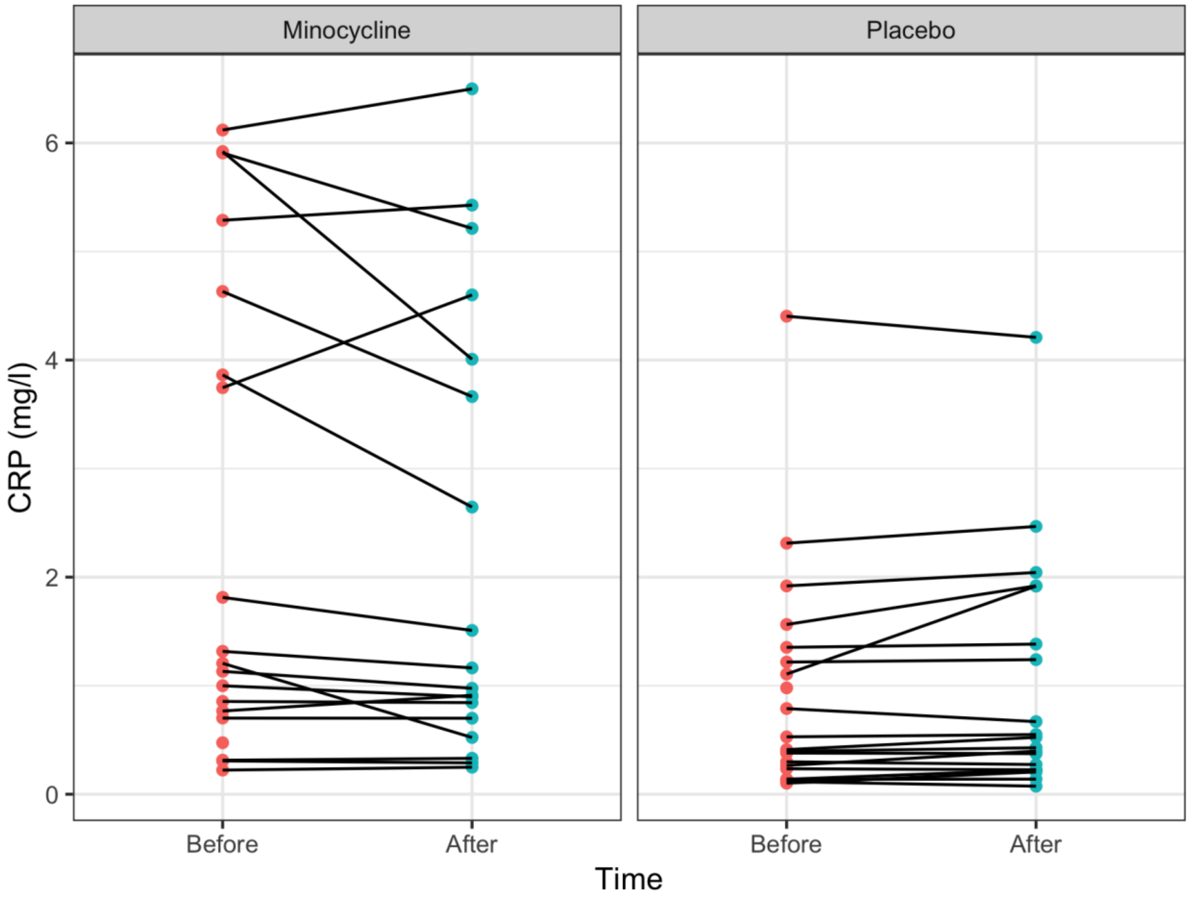

Fig S3 Actual serum CRP concentration before and four hours after minocycline/placebo administration. The CRP concentration of all participants were within what is considered the healthy range (< 10mg/L), which is detectable only by a high sensitivity kit. While the minocycline group had higher baseline CRP concentration, this was because a small sub-group had slightly elevated baseline CRP (2-6mg/L) which skewed the average. The majority of participants had low CRP levels (< 2mg/L) at both time-points. We also get the same result (significant CRP difference between treatment groups after drug administration normalized to baseline levels) after excluding all participants with baseline CRP > 2 mg/L (Minocycline group: n = 11, mean = 0.927, SEM = 0.0596; Placebo group: n = 17, mean = 1.175, SEM = 0.0858; difference between groups after minocycline/placebo administration = 0.248, SEM = 0.117, p = 0.0447) . Slight elevations in CRP levels could be a result of lifestyle factors, such as exercise and smoking [14].

Fig S4: Plasma CRP and cortisol levels

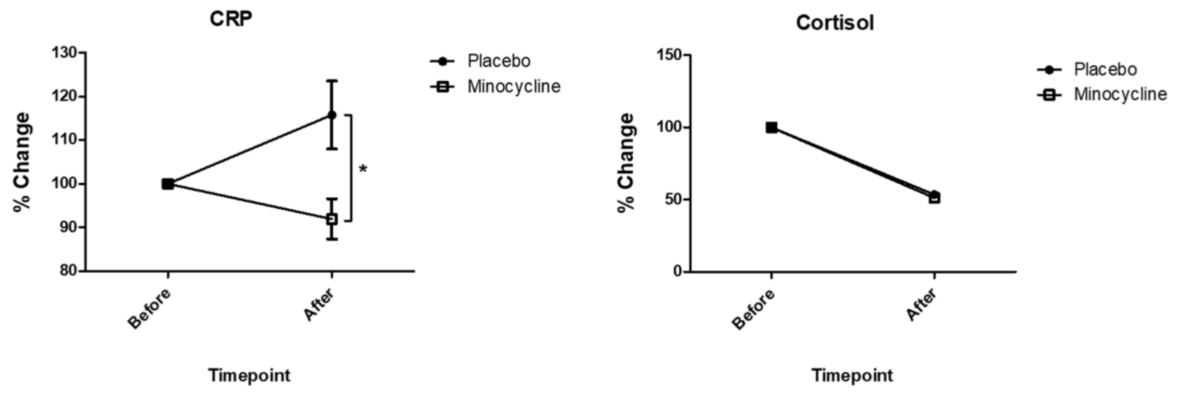

Fig S4 Plasma CRP and cortisol levels before and after placebo/minocycline administration expressed as a percentage change of before. Inflammatory cytokines (IL-1β, TNFα, IL-6) were also measured but as levels were low in healthy volunteers, cytokines measured were largely beyond the detection limit of the kit and comparisons were not meaningful.

*$p < 0.05$

Fig S5: Baseline Metabolite Validation

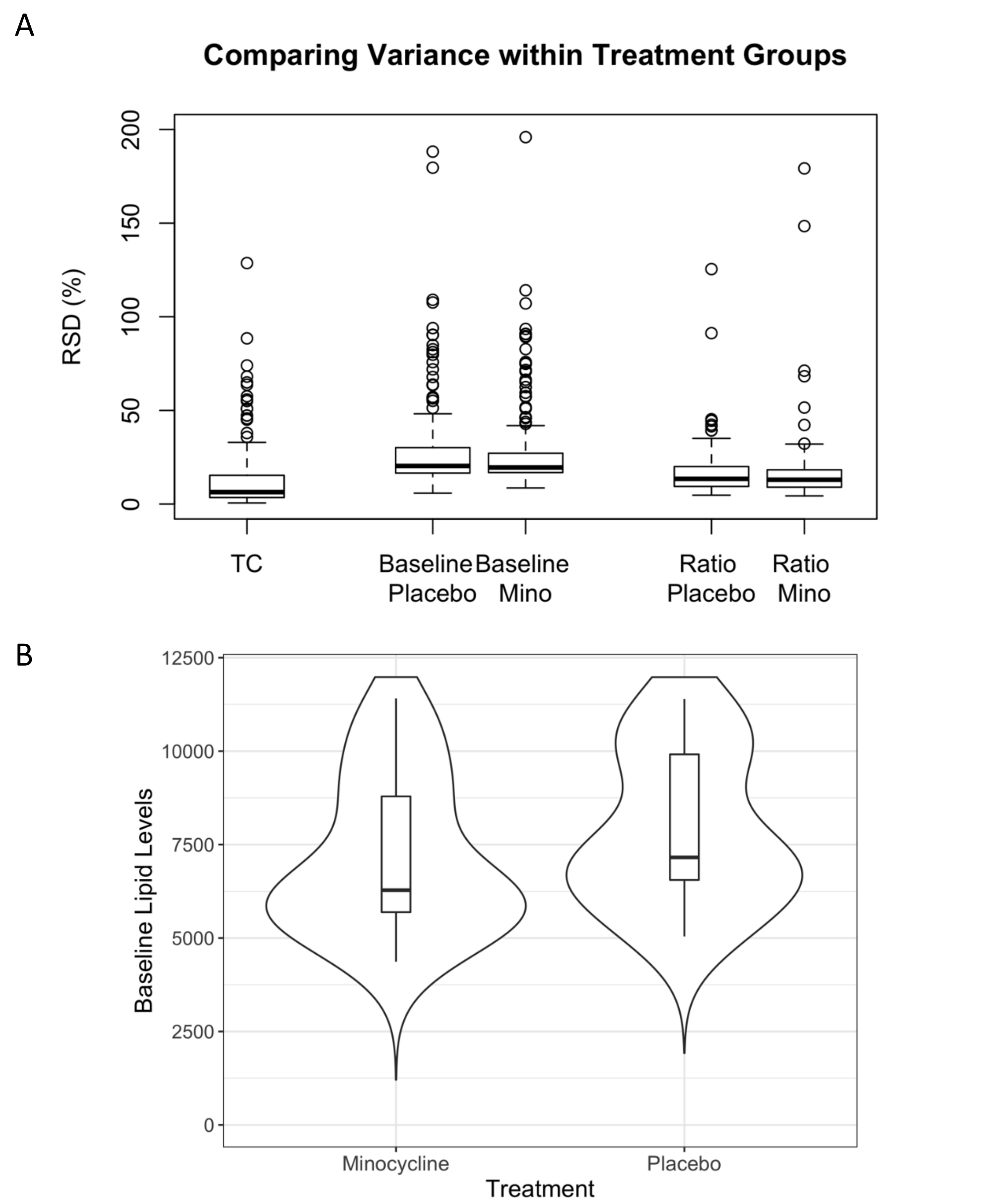

Fig S5 (A) Spectrum wide relative standard deviations (RSD) for technical controls (TC), baseline placebo and minocycline, and the ratio of change (After/Baseline) for both treatment groups. Median RSD values are a commonly used marker of inter-individual variation within datasets [15], and were similar between groups at both baseline and the ratio of change. As expected, TCs, the same sample interspersed throughout the NMR session, had the lowest median RSD values. Median RSD levels were similar to what has been shown in literature for human serum samples.

(B) Lipid levels (calculated by averaging lipid peak bins) had a similar distribution between minocycline and placebo groups at baseline.

Fig S6: Difference (%) in total sum area of all metabolite peaks

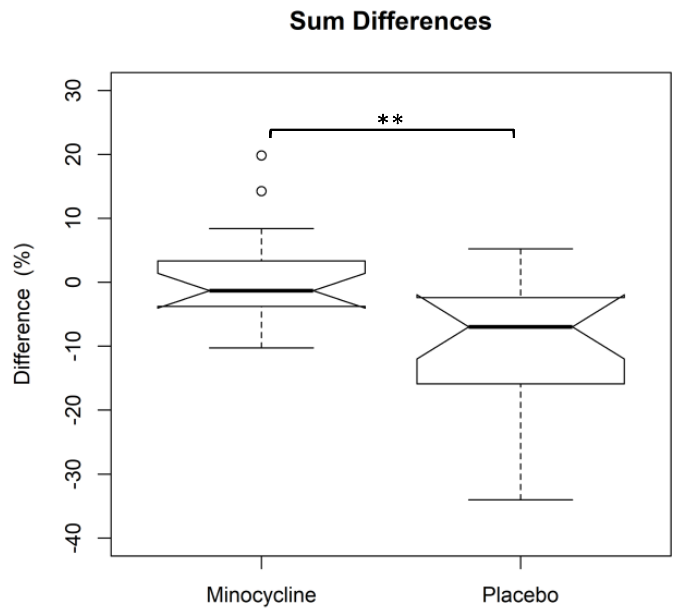

Fig S6 Difference in total sum area of all metabolite peaks between time-point 1 (baseline) and time-point 3 (end of study) expressed as a percentage of time-point 1. The placebo group had decreased serum metabolites at the second time-point. This was expected as participants fasted during the testing visit, and the first blood collection time-point was taken approximately 1 hour after participants ate breakfast, while the second time-point was taken approximately 6 hours postprandial. Thus, we expected that serum metabolite levels would be high the first time-point (1 hour postprandial) and lower at the second time-point (approximately 6 hours postprandial). This is supported by glucose literature that show that blood glucose concentration peaks 1 hour after the start of meal, and returns to preprandial levels within 3 hours [16].

**p < 0.01

Fig S7: Minocycline/Placebo models compared to null models

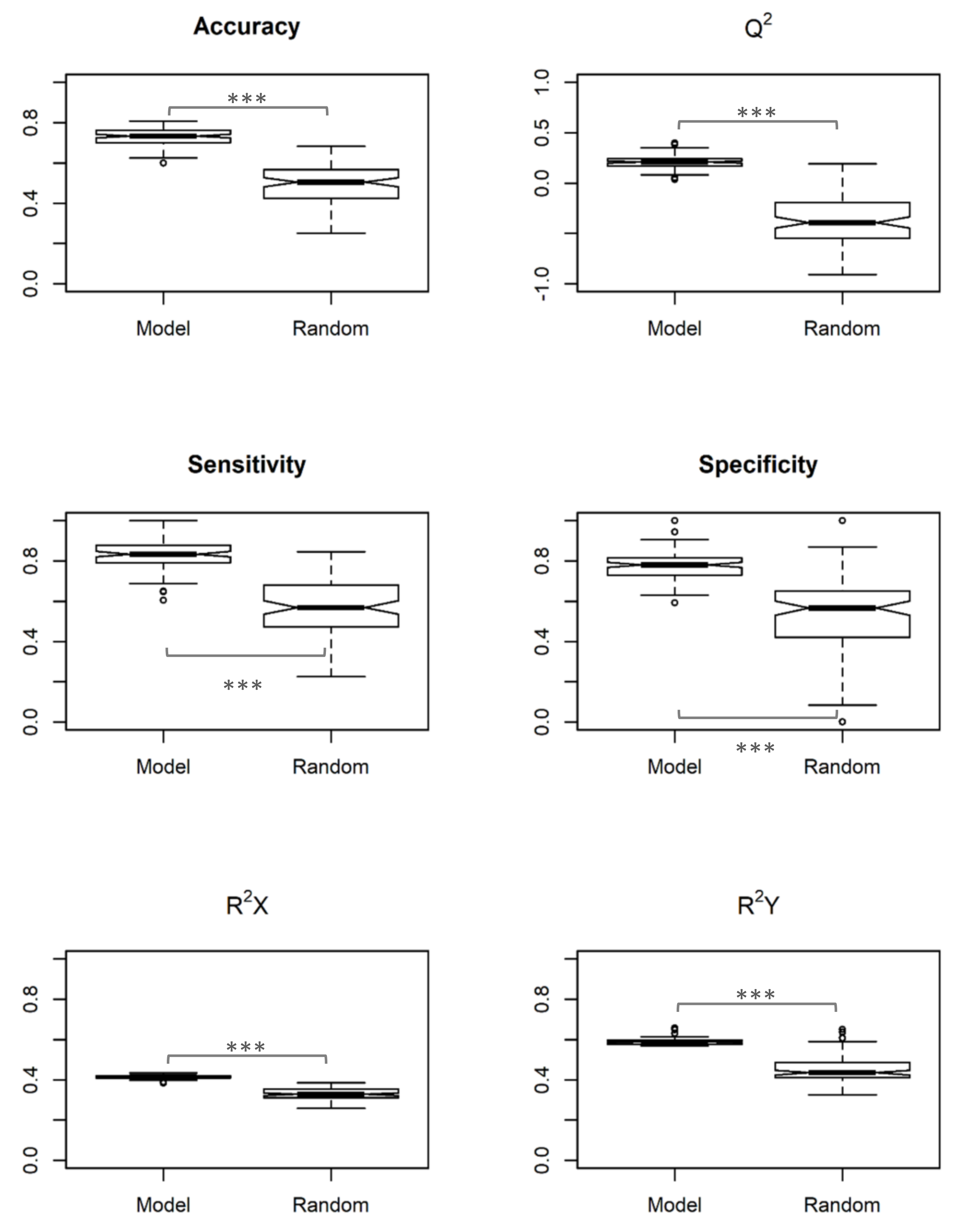

Fig S7 Boxplots comparing between the Minocycline/Placebo models (Model) and null models with randomly permuted group labels (Random). On all counts (Accuracy, Q^2, Sensitivity, Specificity, R^2X, R^2Y), the Minocycline/Placebo models were significantly better. ***$p < 0.001$

Table S1: Demographic data and baseline scores

Table S1 Demographics and baseline data of participants are similar between treatment groups.

	**Minocycline Group N=20**	**Placebo Group N=20**	**Significance (*p* value)**
Gender	10M/10F	10M/10F	1.0
Age (Years)	27.50 (4.56)	27.50 (6.89)	0.464
BMI	23.84 (3.06)	23.31 (1.79)	0.797
NART	115.95 (6.63)	114.62 (4.43)	0.459
STAI (Trait)	31.55 (5.83)	31.90 (7.28)	0.849
BDI	1.30 (2.32)	2.10 (3.74)	0.673
EPQ: Neuroticism	4.15 (2.92)	5.40 (3.97)	0.264
EPQ: Psychoticism	2.65 (1.81)	3.05 (2.50)	0.711
EPQ: Extraversion	14.00 (4.22)	14.30 (4.11)	0.821
EPQ: Lie	9.75 (3.77)	11.60 (3.72)	0.126

Values represent the mean with standard deviation in parentheses.

BMI: Body mass index; NART: National Adult Reading Test; STAI: State-trait Anxiety Index; BDI: Beck Depression Inventory; EPQ: Eysenck Personality Questionnaire

Table S2 Self-reported state changes assessed at three time-points were similar between treatment groups.

	**Minocycline Group**			**Placebo Group**		
	Pre-treatment	Post-treatment 1	Post-treatment 2	Pre-treatment	Post-treatment 1	Post-treatment 2
STAI (State)	28.8 (5.56)	26.8 (6.11)	31.2 (7.16)	27.35 (6.81)	24.8 (5.76)	28.35 (8.63)
PANAS: Positive	32.15 (6.78)	32.8 (8.10)	31.60 (8.34)	33.45 (6.88)	37.55 (5.42)	35.25 (5.83)
PANAS: Negative	12.45 (3.99)	11.00 (2.27)	12.35 (5.14)	12.15 (3.10)	10.95 (1.73)	11.60 (2.26)
BFS: Energy	2.50 (3.93)	3.20 (5.77)	5.85 (6.39)	2.20 (3.62)	2.05 (2.86)	4.60 (5.48)
BFS: Mood	8.25 (10.99)	7.40 (12.00)	10.40 (13.49)	6.00 (5.33)	5.10 (5.19)	7.15 (11.63)
BFS: Total	10.750 (14.54)	10.60 (16.88)	16.25 (18.79)	8.20 (8.29)	7.15 (6.87)	11.75 (15.82)
VAS: Calmness	24.70 (16.63)	25.30 (15.64)	28.35 (18.03)	22.23 (17.02)	24.85 (17.33)	21.60 (16.89)
VAS: Contentedness	20.17 (14.81)	19.68 (12.73)	21.04 (14.97)	18.62 (15.14)	16.81 (11.79)	17.86 (14.06)
VAS: Vigilance	22.35 (13.41)	29.87 (18.13)	33.01 (18.19)	24.93 (14.13)	24.74 (14.68)	31.73 (16.46)

Values represent the mean with standard deviation in parentheses.

STAI: State-trait Anxiety Index; PANAS: Positive and Negative Affective Scale; BFS: Befindlichkeits Scale; VAS: Bond-Lader Visual Analogue Scale

Supplementary references:

1. Ekman, P. and W.V. Friesen, *Pictures of Facial Affect*. 1976: Consulting psychologists Press.
2. Grier, J.B., *Nonparametric indexes for sensitivity and bias: computing formulas* Psychological Bulletin, 1971. **75**(6): p. 424-429.
3. Anderson, N.H., *Likableness ratings of 555 personality-trait words.* J Pers Soc Psychol, 1968. **9**(3): p. 272-9.
4. Matsumoto, D. and P. Ekman, *Japanese and Caucasian facial expressions of emotion (JACFEE).* Intercultural and Emotion Research Laboratory, Department of Psychology, San Francisco State University, CA 1988.
5. Chun, M.M., *Contextual cueing of visual attention.* Trends in Cognitive Sciences, 2000. **4**(5): p. 170-178.
6. Chun, M.M. and Y. Jiang, *Contextual cueing: implicit learning and memory of visual context guides spatial attention.* Cogn Psychol, 1998. **36**(1): p. 28-71.
7. Klinge, C., et al., *Methylphenidate enhances implicit learning in healthy adults.* J Psychopharmacol, 2018. **32**(1): p. 70-80.
8. Harvey, P.O., et al., *Cognitive control and brain resources in major depression: an fMRI study using the n-back task.* Neuroimage, 2005. **26**(3): p. 860-9.
9. Jurynczyk, M., et al., *Metabolomics reveals distinct, antibody-independent, molecular signatures of MS, AQP4-antibody and MOG-antibody disease.* Acta Neuropathologica Communications, 2017. **5**(1): p. 95.
10. R Core Team, *R: A language and environment for statistical computing.*, in *R Foundation for Statistical Computing, Vienna, Austria.* 2016.
11. Thévenot, E.A., et al., *Analysis of the Human Adult Urinary Metabolome Variations with Age, Body Mass Index, and Gender by Implementing a Comprehensive Workflow for Univariate and OPLS Statistical Analyses.* Journal of Proteome Research, 2015. **14**(8): p. 3322-3335.
12. Misra, D. and U. Bajpai, *Metabolite characterization in serum samples from normal healthy human subjects by 1H and 13C NMR spectroscopy.* Bull. Chem. Soc. Ethiop, 2009. **23**(2): p. 211-221.
13. Wishart, D.S., et al., *HMDB 3.0--The Human Metabolome Database in 2013.* Nucleic Acids Res, 2013. **41**(Database issue): p. D801-7.
14. Kushner, I., D. Rzewnicki, and D. Samols, *What does minor elevation of C-reactive protein signify?* Am J Med, 2006. **119**(2): p. 166.e17-28.
15. Parsons, H.M., et al., *Spectral relative standard deviation: a practical benchmark in metabolomics.* Analyst, 2009. **134**(3): p. 478-85.
16. *Postprandial blood glucose. American Diabetes Association.* Diabetes Care, 2001. **24**(4): p. 775-8.
